# Artifact suppression for breast specimen imaging in micro CBCT using deep learning

**DOI:** 10.1186/s12880-024-01216-5

**Published:** 2024-02-06

**Authors:** Sorapong Aootaphao, Puttisak Puttawibul, Pairash Thajchayapong, Saowapak S. Thongvigitmanee

**Affiliations:** 1 Faculty of Medicine, Prince of Songkla University, Songkhla, Thailand; 2 Medical Imaging System Research Team, Assistive Technology and Medical Devices Research Group, National Electronics and Computer Technology Center, National Science and Technology Development Agency, Pathum Thani, Thailand; 3 National Science and Technology Development Agency, Pathum Thani, Thailand

**Keywords:** Cone-beam CT, Iterative reconstruction, Scattering radiation, Metal artifact, Truncation artifact, Sparse-view sinogram, Deep learning

## Abstract

**Background:**

Cone-beam computed tomography (CBCT) has been introduced for breast-specimen imaging to identify a free resection margin of abnormal tissues in breast conservation. As well-known, typical micro CT consumes long acquisition and computation times. One simple solution to reduce the acquisition scan time is to decrease of the number of projections, but this method generates streak artifacts on breast specimen images. Furthermore, the presence of a metallic-needle marker on a breast specimen causes metal artifacts that are prominently visible in the images. In this work, we propose a deep learning-based approach for suppressing both streak and metal artifacts in CBCT.

**Methods:**

In this work, sinogram datasets acquired from CBCT and a small number of projections containing metal objects were used. The sinogram was first modified by removing metal objects and up sampling in the angular direction. Then, the modified sinogram was initialized by linear interpolation and synthesized by a modified neural network model based on a U-Net structure. To obtain the reconstructed images, the synthesized sinogram was reconstructed using the traditional filtered backprojection (FBP) approach. The remaining residual artifacts on the images were further handled by another neural network model, ResU-Net. The corresponding denoised image was combined with the extracted metal objects in the same data positions to produce the final results.

**Results:**

The image quality of the reconstructed images from the proposed method was improved better than the images from the conventional FBP, iterative reconstruction (IR), sinogram with linear interpolation, denoise with ResU-Net, sinogram with U-Net. The proposed method yielded 3.6 times higher contrast-to-noise ratio, 1.3 times higher peak signal-to-noise ratio, and 1.4 times higher structural similarity index (SSIM) than the traditional technique. Soft tissues around the marker on the images showed good improvement, and the mainly severe artifacts on the images were significantly reduced and regulated by the proposed. method.

**Conclusions:**

Our proposed method performs well reducing streak and metal artifacts in the CBCT reconstructed images, thus improving the overall breast specimen images. This would be beneficial for clinical use.

## Background

For breast-conserving surgery, a radiologist localizes a position of abnormal calcifications or masses on a patient’s breast using a thin guide wire or a metal needle before operation. Once a breast specimen is resected from a patient, a verification method to confirm the complete removal of a breast tumor is usually desirable. Traditionally, 2D specimen images acquired from a mammography machine are commonly used for calcification only; however, they are not used in the case of lesions and masses. Cone-beam computed tomography (CBCT) scanners have been widely used in dental, maxillofacial, and orthopedic applications due to accurate 3D data for diagnosis and treatment plans. The use of CBCT has been expanded into breast specimen imaging to verify a tumor free margin [1–3]. Available micro-focused CBCT scanners for high-quality breast specimen imaging include Bruker Skyscan [1–2], and our in-house CBCT named MiniiScan [3]. Even though 3D images from a micro CBCT can provide good image quality, the long acquisition processing times may not be practical for clinical use in a surgery room. This is due to the limitation of X-ray power and high-resolution acquisition. Specifically, the main limitation of the micro-focused X-ray source is the low tube current output, the heat accumulation while scanning, and the cooling time requirement. Therefore, the CBCT image acquisition system must be managed to compensate between noise in reconstructed images and scan times. A common technique to overcome the low tube current and reduce noise is a multi-scan method in projection data. When the number of multi-scans is increased, the noise in the projection images can be continuously reduced. While this method can provide a good signal in the images, the scan time is still increased. The challenging problem is to reduce the scan time while maintaining the image quality on the reconstructed images. One simple solution is to reduce the number of projections by down-sampling along the angles in a sinogram, but this introduces additional streak artifacts in the image. Furthermore, to assist a surgeon before surgery, the abnormal lesion and mass in the patient’s breast are located by a radiologist using a metal needle marker. Then, the breast specimen is resected from the patient and still contains the needle marker. When taking a CT scan of that breast specimen, the corresponding reconstructed images show severe metal artifacts.

Many researchers have published the reduction of artifacts on the reconstructed images [4–10]. Brooks et al. 1978 [4] proposed an up-sampled method using the direction of angles in a sinogram and performed a linear interpolation technique to reduce streak artifacts. Kostler et al. 2006 [5] used non-linear interpolation to synthesize a value in the sinogram from a simulated phantom. Both techniques can provide good results in terms of reducing streak artifacts on the images, but some details are difficult to restore due to the proposed mathematical model. Si Li et al. 2014 [6] proposed a dictional learning-based inpainting method to estimate missing data in the sinogram, which outperformed interpolation. Hoyeon Lee et al. 2018 [7] proposed a solution in the era of machine learning by using deep learning to synthesize missing data. They used a structure of modified U-net model to keep the detail of the sinogram. Using the trained model, this solution can provide better results with accurate synthetization. In the case of a metal artifact, a traditional method is based on the extraction of metal objects from the sinogram. Sorapong Aootaphao et al. 2008 [8] proposed a technique for extracting the metal objects in the sinogram and used a linear interpolation with iterative reconstruction. This technique can reduce metal artifacts effectively, but the results also proposed some distortion of metal objects and soft tissues around metal objects. Muhamud et al. 2020 [9] proposed deep learning for metal artifact reduction. They used the method of metal extraction to obtain the metal tracks in the sinogram and synthesized the metal tracks by using a neural network. Hossein Arabi et al. 2021 [10] published a corresponding work in which they proposed metal artifact reduction in the sinogram and image domains using deep learning with simulated datasets for training a neural network model. Ketcha et al. 2021 [11] proposed another metal artifact reduction in low-dose imaging using two neural network models on both sinogram and image domains. Although their results on cadavers showed remarkable artifact reduction, the contrast in some areas of the reconstructed images was lost.

In this work, we proposed a method that can alleviate streak and metal artifacts on reconstructed images using deep learning. Two neural network models [7, 11, 12] were used to improve image quality in both sinogram and image domains. While training the neural network models, a pair of noisy and ground truth datasets must be provided to the model. Unfortunately, those pairs of datasets cannot be obtained simultaneously in real datasets, thus artifact datasets were simulated from the ground truth [10] for training the model. The real datasets containing metal objects were used for testing, and all results from the proposed work were compared to other techniques.

## Material and method

### Structure of the proposed method with deep learning

**Fig. 1:**
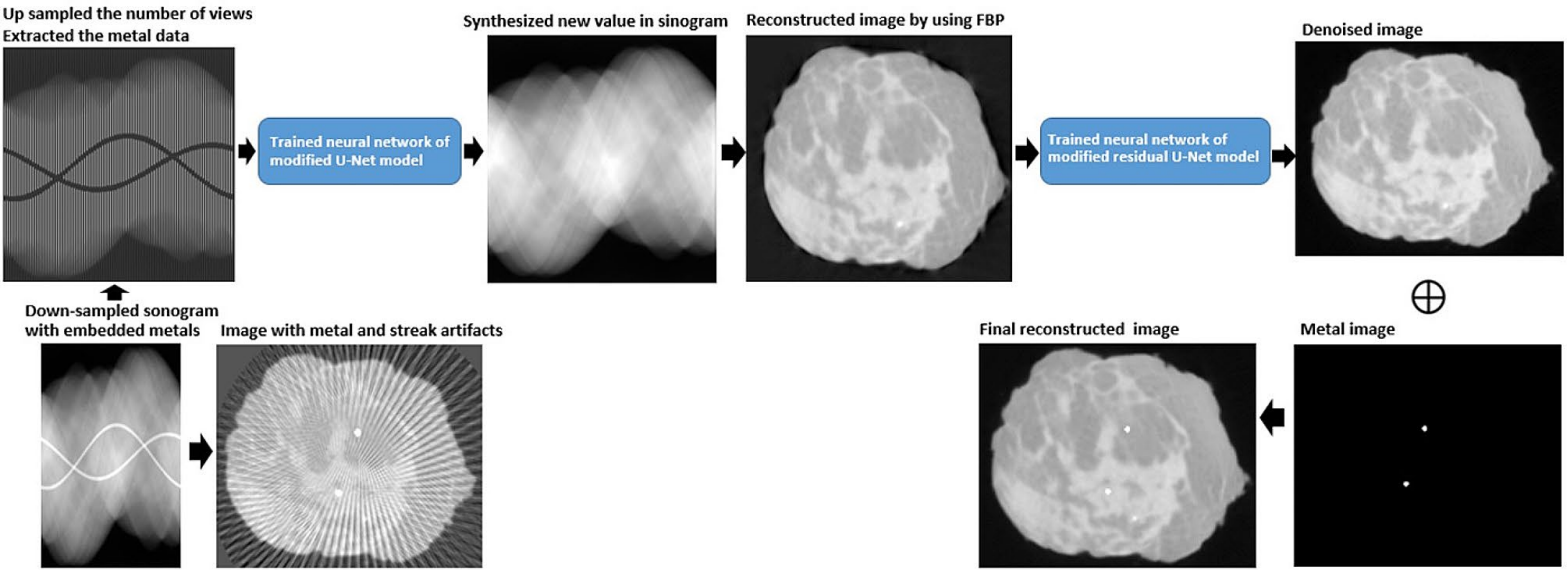
The proposed method with deep learning for reducing streak and metal artifacts

**Fig. 2:**
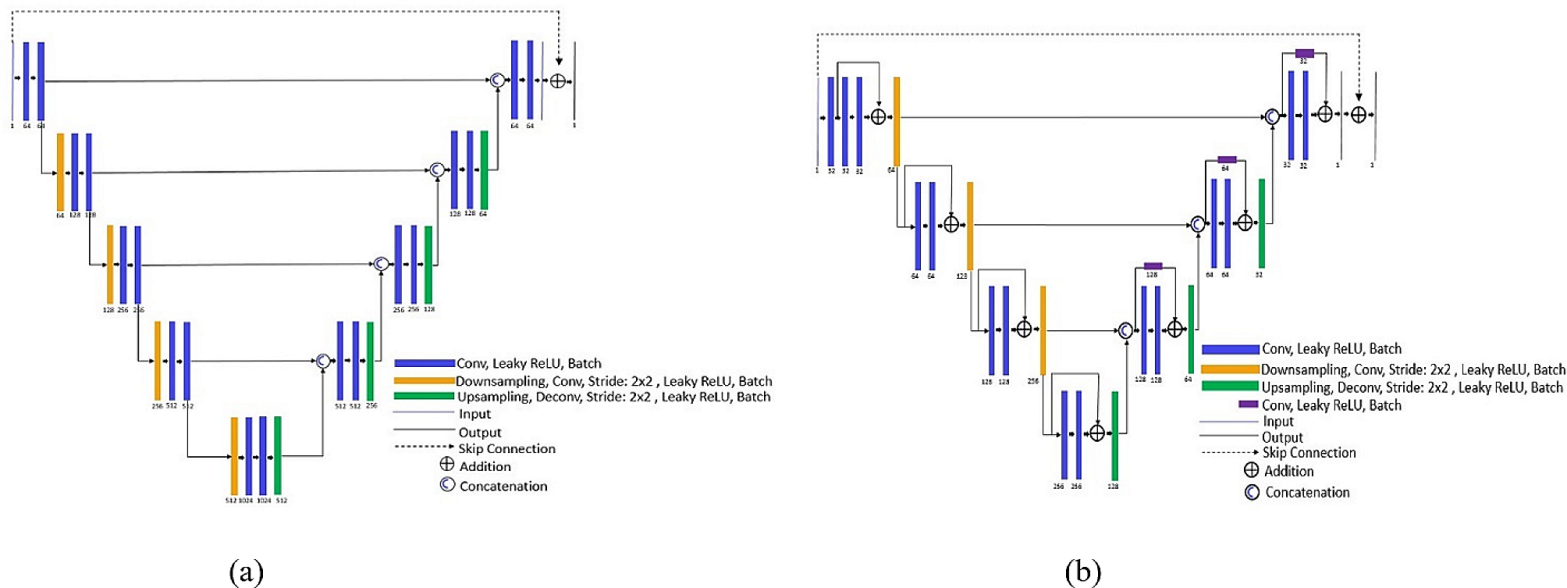
**(a)** The structure of modified U-Net model and **(b)** the structure of modified ResU-Net model

Due to a high performance of neural networks in estimation and prediction, this work established a deep learning method to overcome two main artifacts: streak and metal artifacts. As shown in Fig. 1, a technique for metal artifact reduction (MAR) is proposed using two different neural networks. The modified U-Net [7] model was used for interpolation in the sinogram domain, while another modified Residual U-Net (ResU-Net) [12] model handled the residual noise in the reconstructed images, as shown in Fig. 2. The U-Net model published by Hoyeon Lee [7] was used in this work to synthesize new values in up-sampled views and metal tracks in sinograms. Some positions in this model were modified as a pooling layer and added by a residual learning scheme. Instead of a traditional max-pool filter, a down-sampling technique of the structure’s pooling layers used a convolutional operation with a stride of 2 × 2 pixels to resize the input data passing each layer. In addition, a residual learning scheme technique was provided to accelerate convergence while training the model. For example, the input and final layers in the model were connected by skipping data and the operation of summation was added, as shown in Fig. 2 (a). Similarly, the structure of the ResU-Net model [12] was modified in this work. It used convolution with the stride of 2 × 2 pixels for down sampling the input data, as well as the skip connection. Furthermore, in Fig. 2 (b), this model included the operation of addition across layers in the same stages to maintain the detail of data passing through layers.

### Data acquisition

We proposed the deep learning method to reduce streak and metal artifacts with a reduction of 2D projection images. Clinical datasets of breast specimens were obtained from an in-house micro CBCT scanner (MiniiScan, Thailand) installed in the operation room. This CBCT scanner employs a low-power micro-focus X-ray generator with 50 kVp, 0.063 mAs per projection, and a 15 cm x 15 cm flat panel detector with a pixel pitch of 0.127 mm. Due to the low power, a multi-scan method was used to reduce noise. Four clinical datasets used in the experiment were divided into two groups. Group 1 contained two datasets of metal-free projections for training a neural network model, many two datasets with 720 projections acquired by scanning twice. Group 2 contained two datasets of embedded metal objects for testing and validating the results. The original datasets from CBCT included 360 projections that were averaged from several repeated projection views. Furthermore, the entire clinical datasets for each group were corrected for X-ray scattering [13–15] before proceeding in the proposed method.

In this work, a filtered backprojection (FBP) method [16–17] was primarily used in the proposed work, while an iterative reconstruction (IR) [18–21] was used for comparison in the experiment. The parameters for FBP and IR were appropriately adjusted to allow a fair comparison in image quality. Note that the conventional metal artifact reduction using the FBP with linear-interpolated sinograms is currently implemented in our in-house micro CBCT. Moreover, we also added two more methods using deep learning. The first method is to use deep learning on the reconstructed image only and the second method is to use deep learning on the sinograms only.

In the first method, to obtain the denoised FBP images, the step of interpolation in the sinogram domain with U-Net deep learning like in the proposed approach was ignored, and simple linear interpolation was applied instead to fill in the missing data on the sinogram. The linear-interpolated sinogram was reconstructed by the FBP method, and then the reconstructed images were denoised by ResU-Net. On the other hand, the second method performed interpolation on the sinograms using U-Net, and then the interpolated sinograms were reconstructed to produce the reconstructed images. Here, a further denoising process on the reconstructed images using ResU-Net was ignored.

### Simulation of streak and metal artifacts on sinogram and image domains for training the neural network

Both ground truth and artifact datasets were required for training the neural network model, but unfortunately, the artifacts datasets for training cannot be obtained at the same as the ground truth. Datasets used for training must be in same domain or environment, and they had an only difference as the noise or artifacts. In fact, we cannot acquire the real datasets of patients, which were with and without artifacts in the same environment. Thus, they must be simulated and generated from ground truth. In terms of image simulation of streak artifacts, the reduction of scan time in CBCT can be interpreted as decreased projections or down-sampled sinogram views. To mock the metal artifacts [10], an artifact-free reconstructed image from ground truth can be added by a metallic wire and then forward-projected to obtain a sinogram with embedded metal data, which mimicked the marker in the breast specimen images.

The proposed method with deep learning can handle two artifacts generated from the down-sampled sinogram with the embedded metal needle. Training a model in a neural network was a critical step, and the model’s performance was dependent on how well the datasets were prepared. When two datasets in Group 1 were prepared for simulation of the artifacts, they contained the ground truth from 360-view sinograms without embedded metals and the reconstructed images. Here, 360 projection images were rearranged to construct sinograms. The sinogram used in the experiment contained 512 × 360 pixels, and the artifact-free reconstructed images contained 300 × 300 × 300 voxels.

To generate metal artifacts, the artifact-free reconstructed images were modified by adding a metallic wire, which mimic a needle marker used in a real breast specimen. The metal wire was embedded into the reconstructed images, and then the metal-embedded images were forward projected to obtain the metal-embedded sinogram. Due to the known scanning geometry, the forward-projected approach followed the cone-beam geometry to produce a sinogram using a line-integral technique based on the Beer’s law [16–17]. The sinograms were simulated at every one degree for 360 degrees around the object. In this experiment, the simulation did not include a beam hardening effect due to the small size of a wire and a breast specimen. Finally, those simulated sinograms with metal were performed by FBP to generate reconstructed images with metal artifacts. Furthermore, the sinograms were down sampled by 4 from 360 to 90 views to simulate the reduction of scan time in CBCT; as a result, the streak and metal artifacts were certainly visible on the reconstructed images. Thus, the simulated embedded-metal sinograms and artifact images were used as the datasets or input data for training the model.

**Fig. 3:**
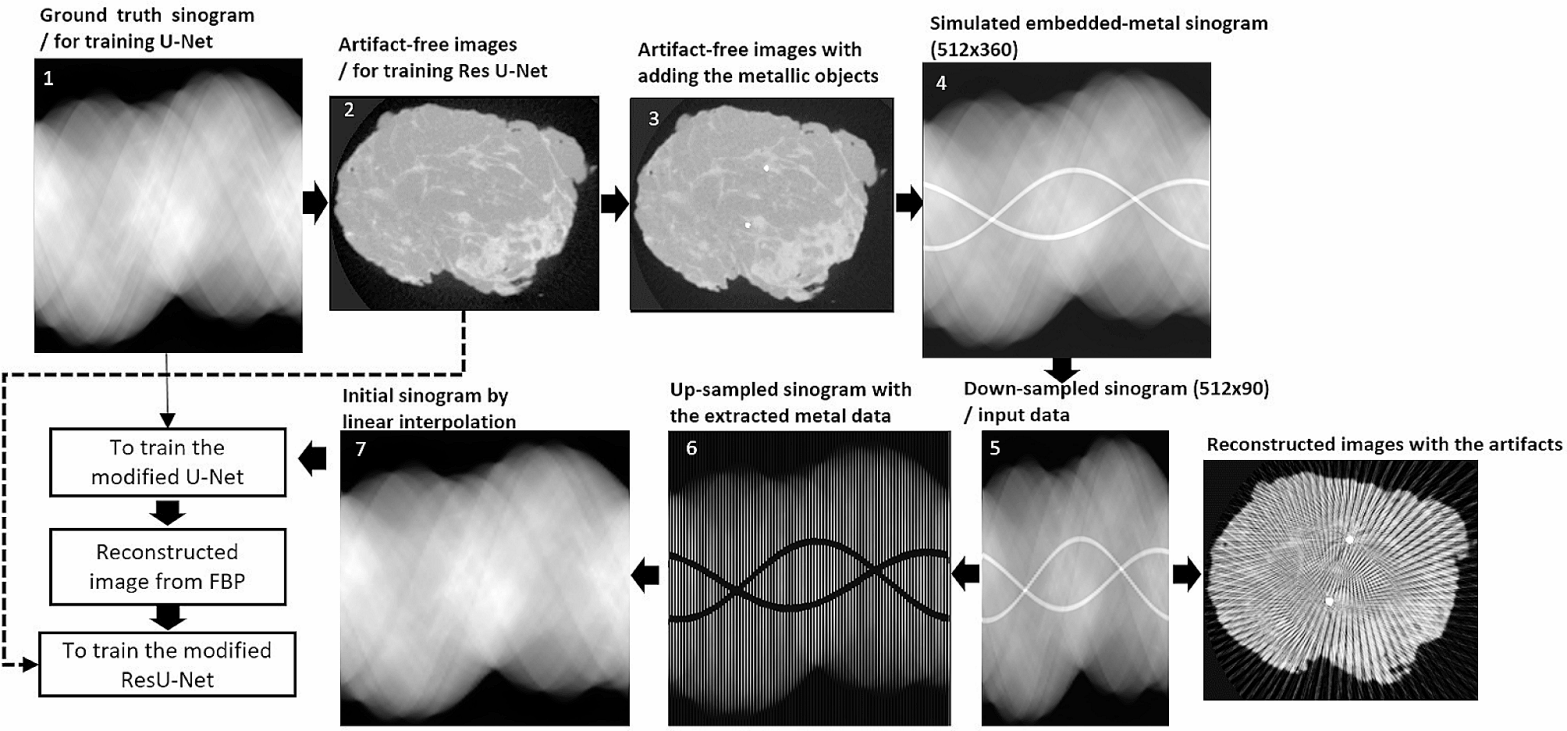
The process to simulate streak and metal artifact datasets for training the model of neural network

The embedded-metal sinograms of 512 × 90 pixels simulated from the previous step were the input data for training. The initial input sinogram was up sampled from 90 to 360 views, and its metal data was also extracted. To estimate the metal tracks in the input sinogram from the metal artifact reconstructed images, the metal data in the reconstructed images were segmented by thresholding to remain only the metal data. Then the metal-only reconstructed images were forward-projected to locate the metal position in the sinogram domain. After upsampling and extracting metal data in the sinogram, the missing data were initially synthesized from linear interpolation. The entire process of simulation can be summarized as shown in Fig. 3.

This work trained the modified U-Net model using the datasets from Group 1 that contained the ground truth and simulated-noisy sinograms. The image patches extracted from the sinogram were chosen by hand and had a size of 64 × 64 pixels. The number of image patches used for training and validation of the model was 180,000 and 18,000, respectively, and an iteration of training was 100 epochs. For stable convergences while training the model, an optimizer in the loss function chose Adam’s methods to measure a root-mean-square error with a learning rate of 0.00001.

For training the modified ResU-Net model, we used pair datasets between the artifact-free reconstructed images from the ground truth sinogram and the reconstructed images from the synthesized sinogram in the previous step (the modified U-Net model). The main feature on the reconstructed images were chosen for training, especially, the features destroyed by the artifacts was emphasized, and they were provided in a set of image patches. The image patches used in this work had a size of 48 × 48 pixels. There were 458,640 image patches extracted from the entire reconstructed images in a 3D matrix used for training, and 80,000 image patches approximated from 18% of training used for validation. Due to limited computation resources in this work, the modified ResU-Net model was trained using 1300 epochs to save time, and an optimizer in the loss function used the Adam’s techniques with the root-mean-square error. Additionally, time in updating parameters while training the modified ResU-Net model was reduced by finding a suitable learning rate of the model. If the learning rate was too very small, the loss function would require a long time to converge. Thus, the learning rate for training the modified ResU-Net model was appropriately increased as the rate of 0.001. Table 1 summarizes all the training parameters.

**Table 1:** Parameter setup for training the model of neural network

	Modified U-Net in sinogram domain	Modified ResU-Net in image domain
Number of image patches for training	180,000	458,640
Number of image patches for validation	18,000	80,000
Size of image patches	64 × 64	48 × 48
Epoch	100	1300
Optimizer	Adam	Adam
Learning rate	0.00001	0.001
Loss Function	RMSE	RMSE

1 $$RMSE = \sqrt {\frac{{\sum\nolimits_{n = 0}^N {{{({m_x} - {m_{ref}})}^2}} }}{N}}$$

### Evaluation method

The reconstructed images with artifact reduction were evaluated in the aspect of image quality and performance of deep learning. In this work, the root mean square error (RMSE), the peak signal-to-noise ratio (PSNR), and the structural similarity index (SSIM) were used to evaluate the performance of the trained model as shown in Eqs. (1)–(3).

2 $$PNSR = 10\,{\log _{10}}\left( {\frac{{m_{peak}^2}}{{MSE}}} \right)$$

where *m*_*x*_ is the average value in the region of interest (ROI), *m*_*ref*_ is the average value of the reference or background in the ROI, and N is the number of the entire pixels in the ROI.

3 $$SSIM = \frac{{\left( {2{m_{ref}}{m_{exp}} + {k_1}} \right)\left( {2{\delta _{ref,exp}} + {k_2}} \right)}}{{\left( {m_{ref}^2 + m_{exp}^2 + {k_1}} \right)\left( {\delta _{ref}^2 + \delta _{exp}^2 + {k_2}} \right)}}$$

where *m*_*peak*_ is the maximum intensity between the expected and ground truth images, and *MSE* is the mean squared error between both images.

where *m*_*exp*_ is the average intensity of the expected images, ${\delta }_{ref}$ and ${\delta }_{exp}$ are the variance of the ground truth and expected images, respectively, and ${\delta }_{ref,exp}$ is the covariance of both images. The defaults k_1_ and k_2_ in SSIM are always defined as 0.001 and 0.03, respectively.

For evaluating image quality on the reconstructed images, the contrast-to-noise ratio is used as follows:

4 $$CNR = \frac{{({m_x} - {m_{ref}})}}{{\sqrt {\left( {\sigma _x^2 + \sigma _{ref}^2} \right)} }}$$

where ${\sigma }_{x}$ is the standard deviation of the ROI. In addition, we plotted the intensity value on the reconstructed images to compare the proposed work with others.

### Experimental results

**Fig. 4:**
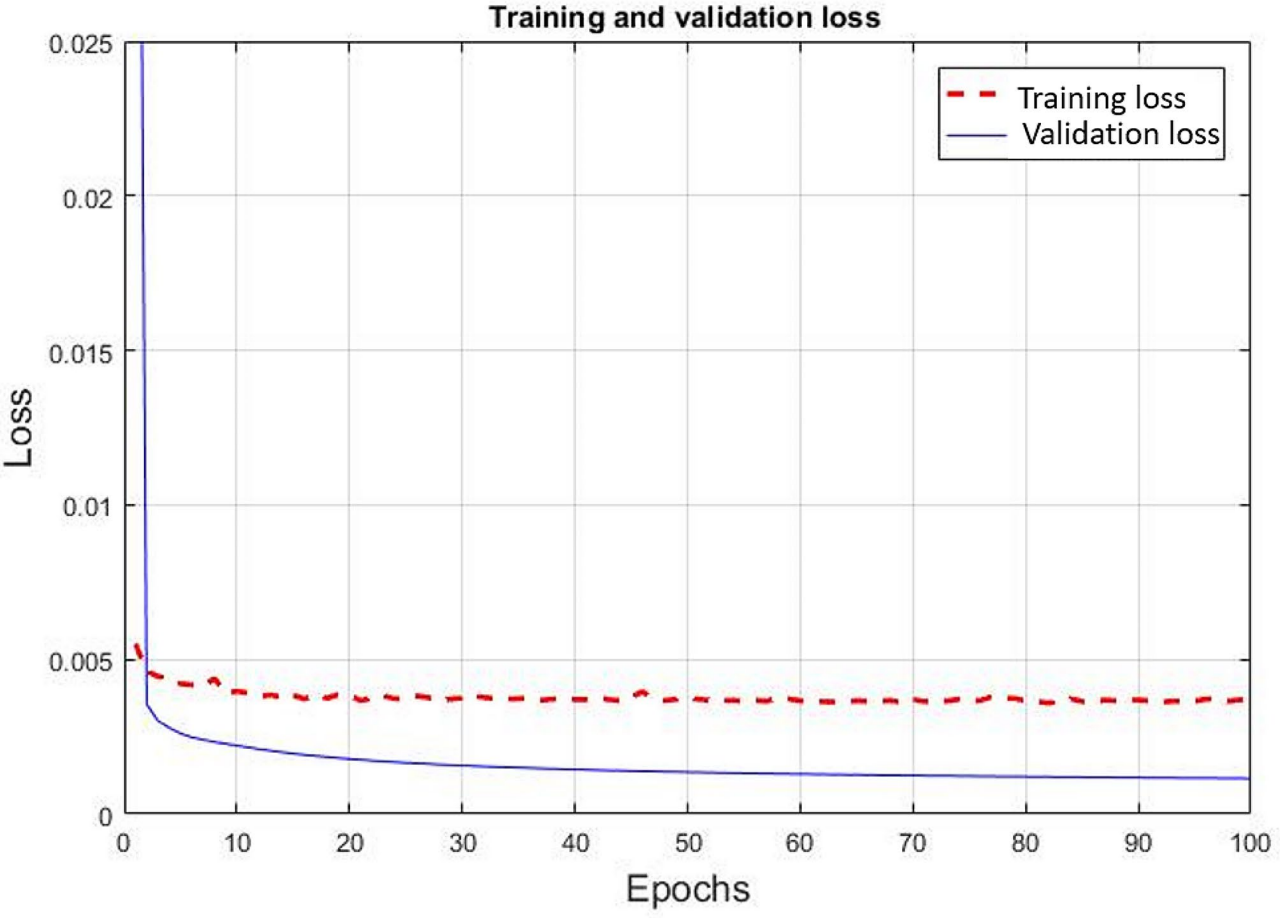
The loss function for training the modified U-Net model

After training the model, the validation and training loss functions can be plotted. The model’s performance can be seen in the stable convergence of validation values, which were drastically reduced and stayed close to the training values, as shown in Fig. 4. From the validation loss, the trained model can provide high performance in synthesizing the accurate value in the sinogram.

**Fig. 5:**
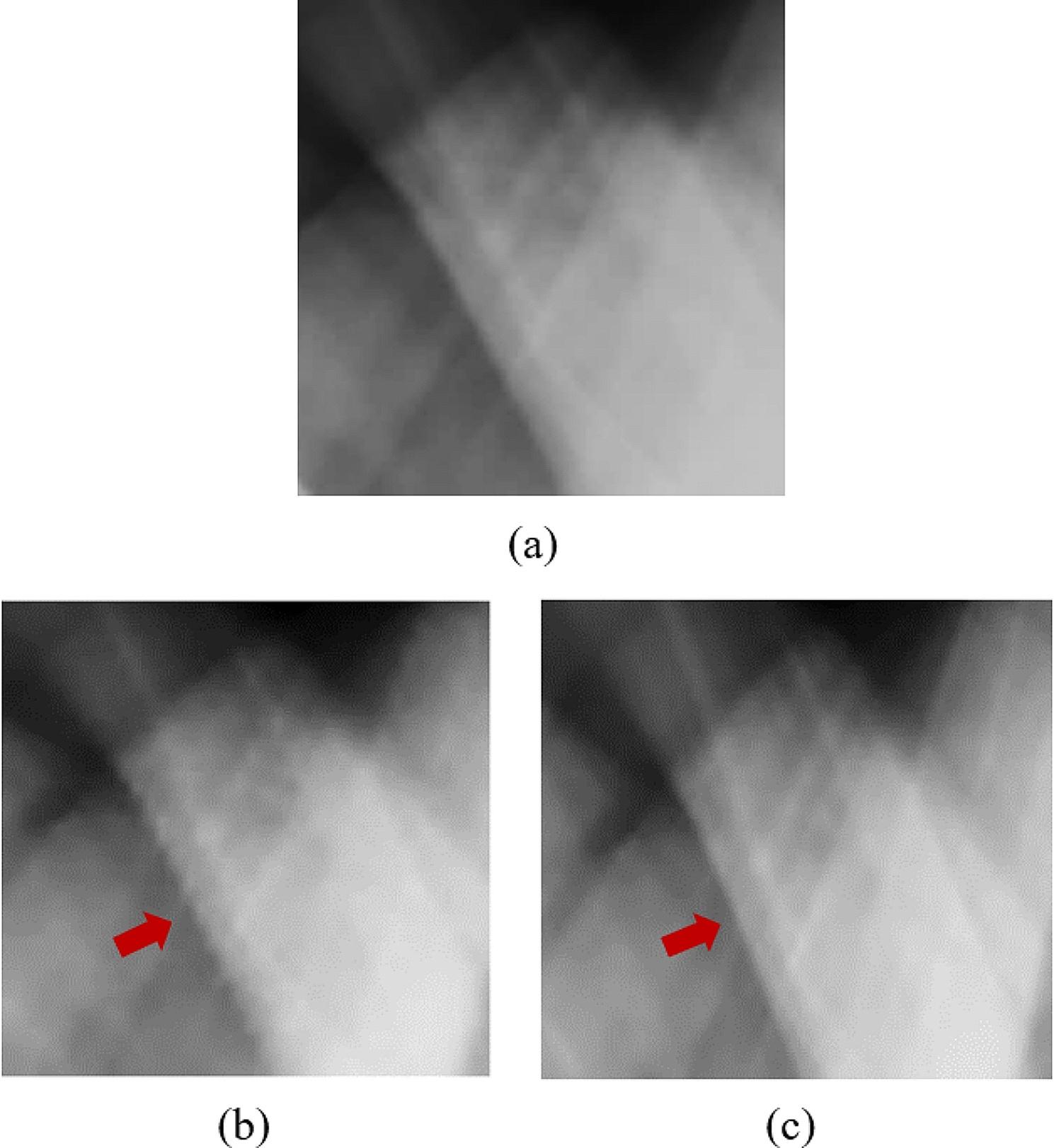
The sinogram results of Case 1 in Group 2: **(a)** the ground truth sinogram, **(b)** the sinogram with linear interpolation, and **(c)** the synthesized sinogram

**Fig. 6:**
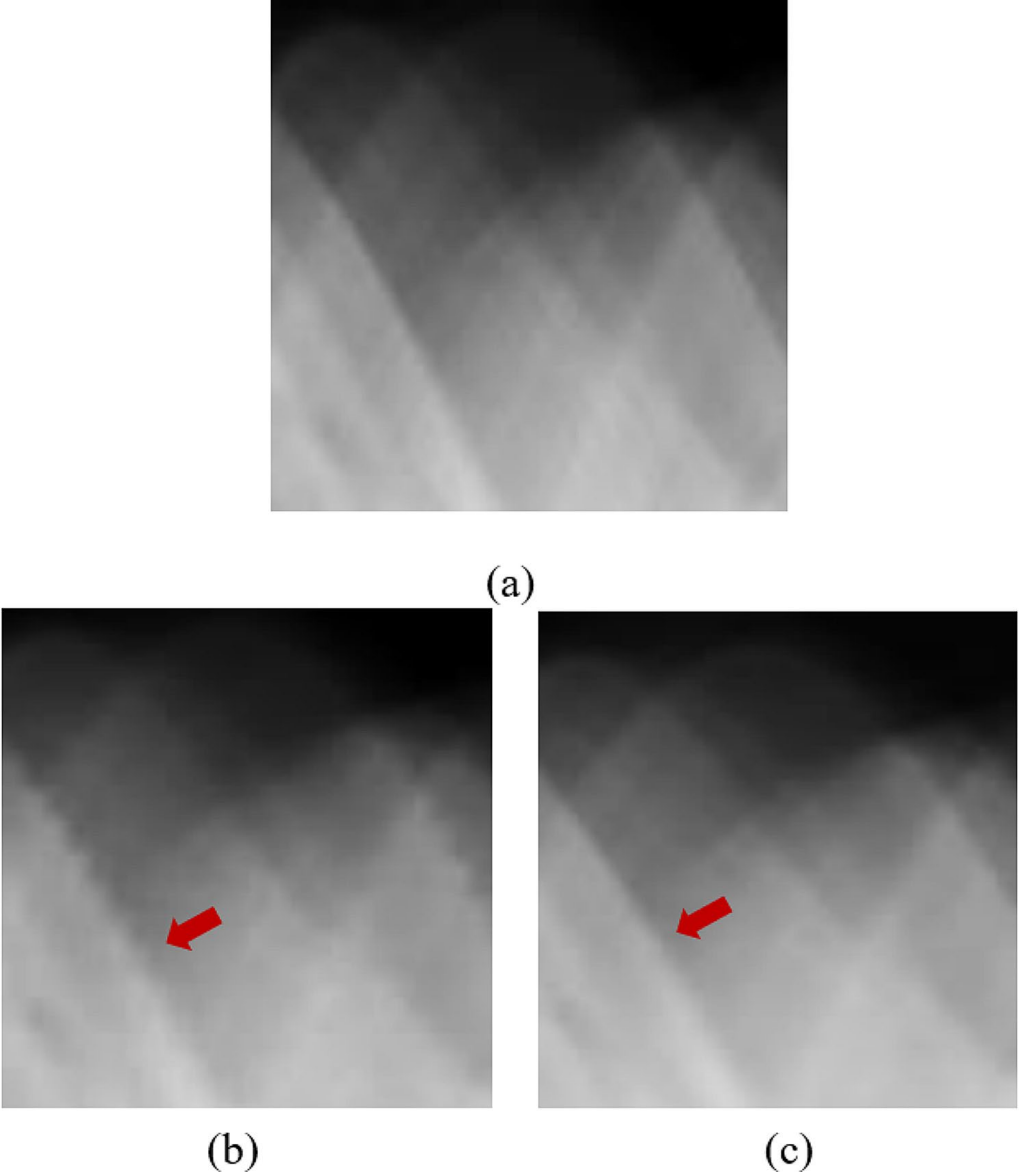
The sinogram results of Case 2 in Group 2: **(a)** the ground truth sinogram, **(b)** the sinogram with linear interpolation, and **(c)** the synthesized sinogram

For testing the trained model, we utilized Group 2 datasets containing two breast specimen cases with embedded metal data in the sinogram. The prepared sinograms from Case 1 were defined by the initial value from linear interpolation as shown in Fig. 5 (b). Then, they were synthesized by the trained modified U-Net model as shown in Fig. 5 (c) and compared to the ground truth sinogram as shown in Fig. 5 (a). The sample improved details in the synthesized sinogram against the linear-interpolated sinogram were shown in the red arrows indicating the edges in the sinogram. Figure 6 shows the results from Case 2 when the up-sampled views and metal positions in the sinogram were replaced by the initial values from linear interpolation (Fig. 6 (b)), followed by the synthesized sinograms from the trained modified U-Net model (Fig. 6 (c)) compared to the ground truth sinogram (Fig. 6 (a)). The performance of the trained model was assessed by SSIM used for comparison and shown in Table 2. Both SSIM values from two breast specimen cases of the modified U-Net model were greater than those of the linear interpolation, where the SSIM values closer to 1 indicate higher image quality. In addition, the error relative to the ground truth was illustrated by the measured RMSE in the ROI, and the noise reduction in the sinogram was measured by PSNR. The RMSEs from both cases were extremely low, and the PSNRs were also higher than those from linear interpolation due to low noise.

**Table 2:** Comparison of SSIM, PSNR and RMSE in the synthesized sinograms using dataset in Group 2

	SSIM	RMSE	PSNR
**Case 1**			
Interpolation	0.9878	0.0055	45.2660
Modified U-Net model	0.9952	0.0030	50.3672
**Case 2**			
Interpolation	0.9762	0.0063	43.9628
Modified U-Net model	0.9863	0.0046	46.7212

**Fig. 7:**
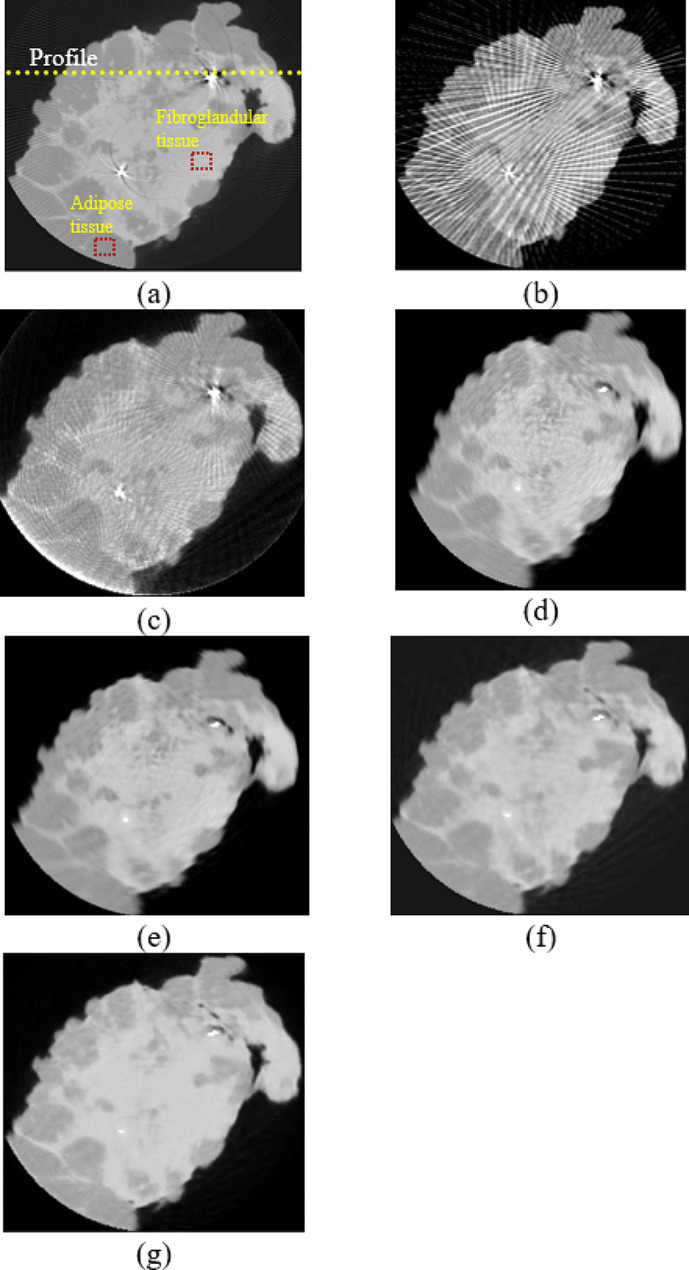
The reconstructed images from different methods: **(a)** the reference image, **(b)** conventional FBP, **(c)** IR, **(d)** FBP with linear-interpolated sinograms, **(e)** denoised FBP with ResU-Net, **(f)** the sinograms with U-Net, and **(g)** the synthesized sinograms with the modified U-Net and ResU-Net models in the proposed method

**Fig. 8:**
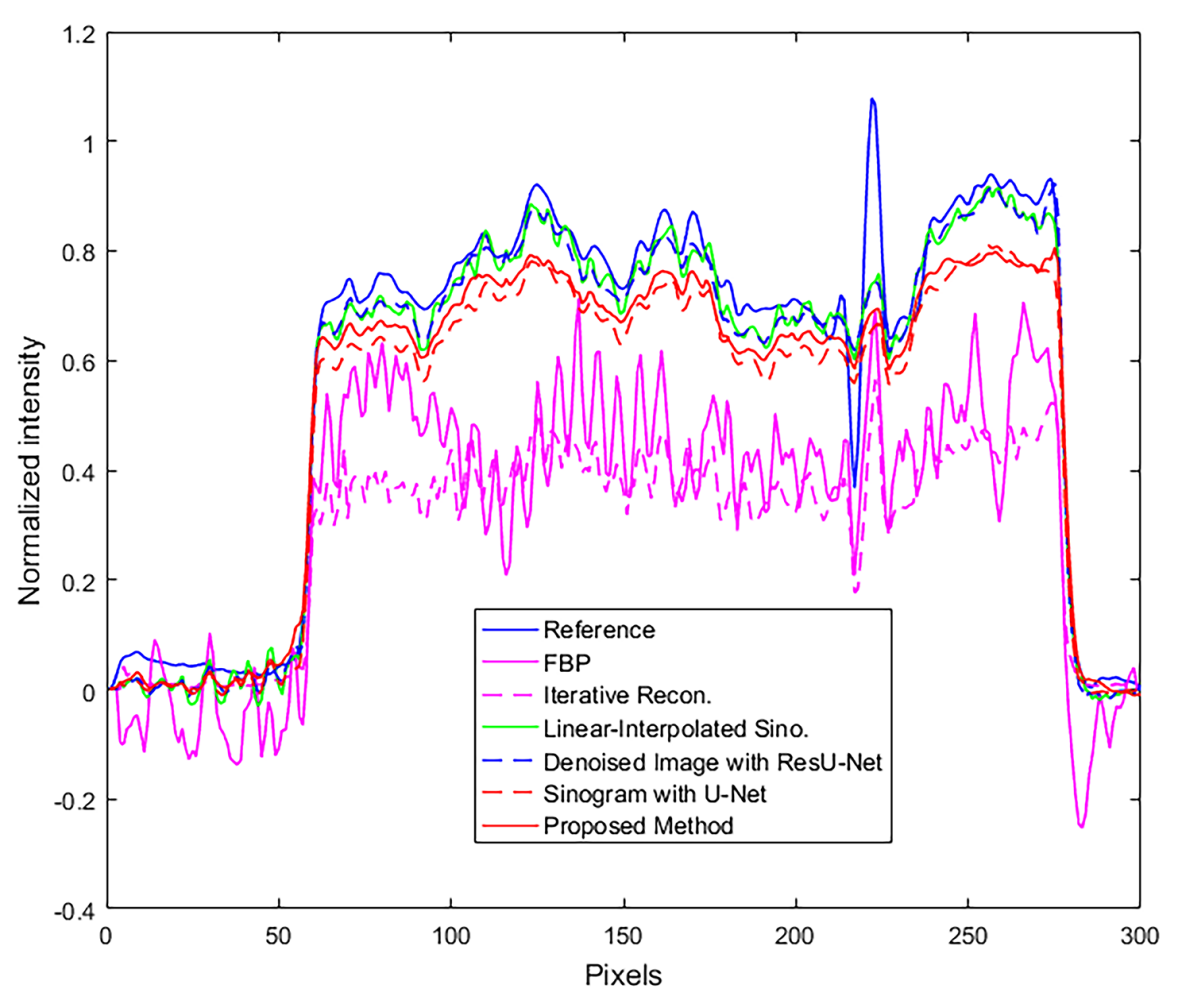
The profiles of the reconstructed images along with the dash-yellow line

From the proposed method with the dataset in Group 2, the completed synthesized sinograms from the modified U-Net model in the previous step were processed continuously and reconstructed by the FBP method to obtain the reconstructed images. The modified ResU-Net model was then applied to the images to reduce the remaining noise. Finally, the denoised images were combined with the metal data to produce the final reconstructed images as shown in Fig. 7. Figure 7 (a) depicted a reference image with metal artifacts or original image. The images from FBP (filter type: shepp logan, cutoff frequency: 1.0) and IR (penalized-likelihood reconstruction, smoothness control = 150, edge preservation = 0.00001, 100 iterations) are shown in Figs. 7 (b) and (c), respectively. In terms of image quality from reconstruction, the streak artifacts in the IR image shown in Fig. 7 (c) were reduced when compared to the image from FBP. Figure 7 (d) shows the cross-section image from FBP with the linear-interpolated sinograms. To compare the proposed method against other deep learning approaches, Figures 7 (e) and (f) depict the denoised FBP image with ResU-Net and the image from the sinogram using U-Net, respectively. Figure 7 (g) shows the image from the proposed method using the modified U-Net and ResU-Net models. The image quality and performance of the models were measured using CNR, PSNR, and SSIM, as shown in Table 3. Those measurements selected the artifact-free areas on the reconstructed images. The areas surrounding the marker needle or lesion in a fibroglandular tissue of the breast specimen were measured, i.e., the CNR was calculated between the fibroglandular and adipose tissues. The SSIM value in the images from the proposed method was higher than that of others, and steak artifacts and noise from the proposed method were also reduced. The CNR and PSNR values from the proposed method were higher than those from FBP, IR, FBP with the linear-interpolated sinograms, denoised FBP with ResU-Net, and FBP with the sinograms using U-Net. Despite the fact that the PSNR from the proposed method was slightly higher than that from the image using linear-interpolated sinograms, the SSIM from the modified ResU-Net model provided a higher value because this model emphasized on reducing the apparent artifacts in the images. The profile plot in Fig. 8 shows that noise in the proposed approach was considerably decreased, and the artifacts, such as overshoot near the metal object, were significantly reduced when compared to other profiles.

**Fig. 9:**
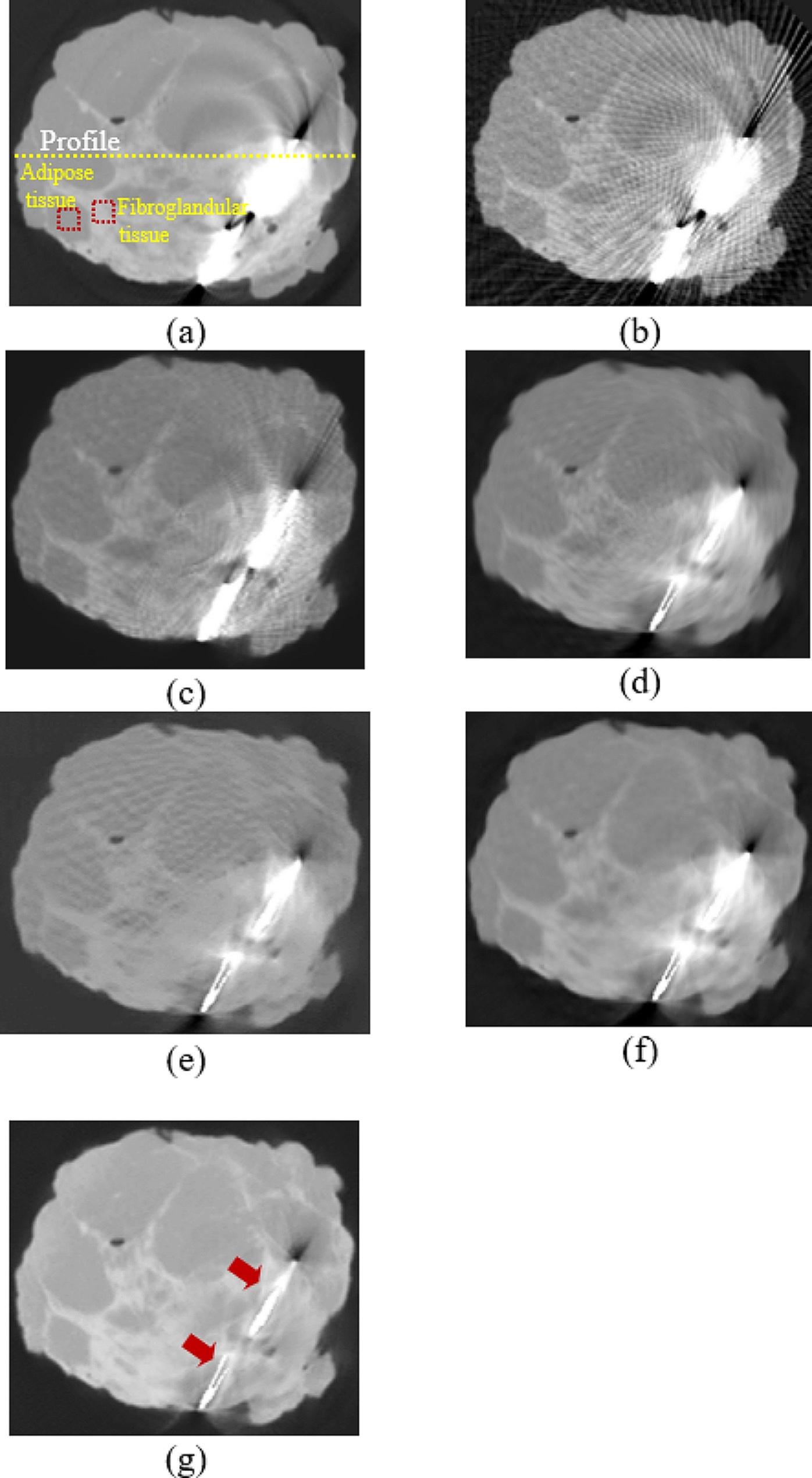
The reconstructed images from different methods: **(a)** reference image, **(b)** conventional FBP, **(c)** IR, **(d)** FBP with linear-interpolated sinograms, **(e)** denoised FBP with ResU-Net, **(f)** the sinograms with U-Net, and **(g)** the synthesized sinograms with the modified U-Net and ResU-Net models in the proposed method

**Fig. 10:**
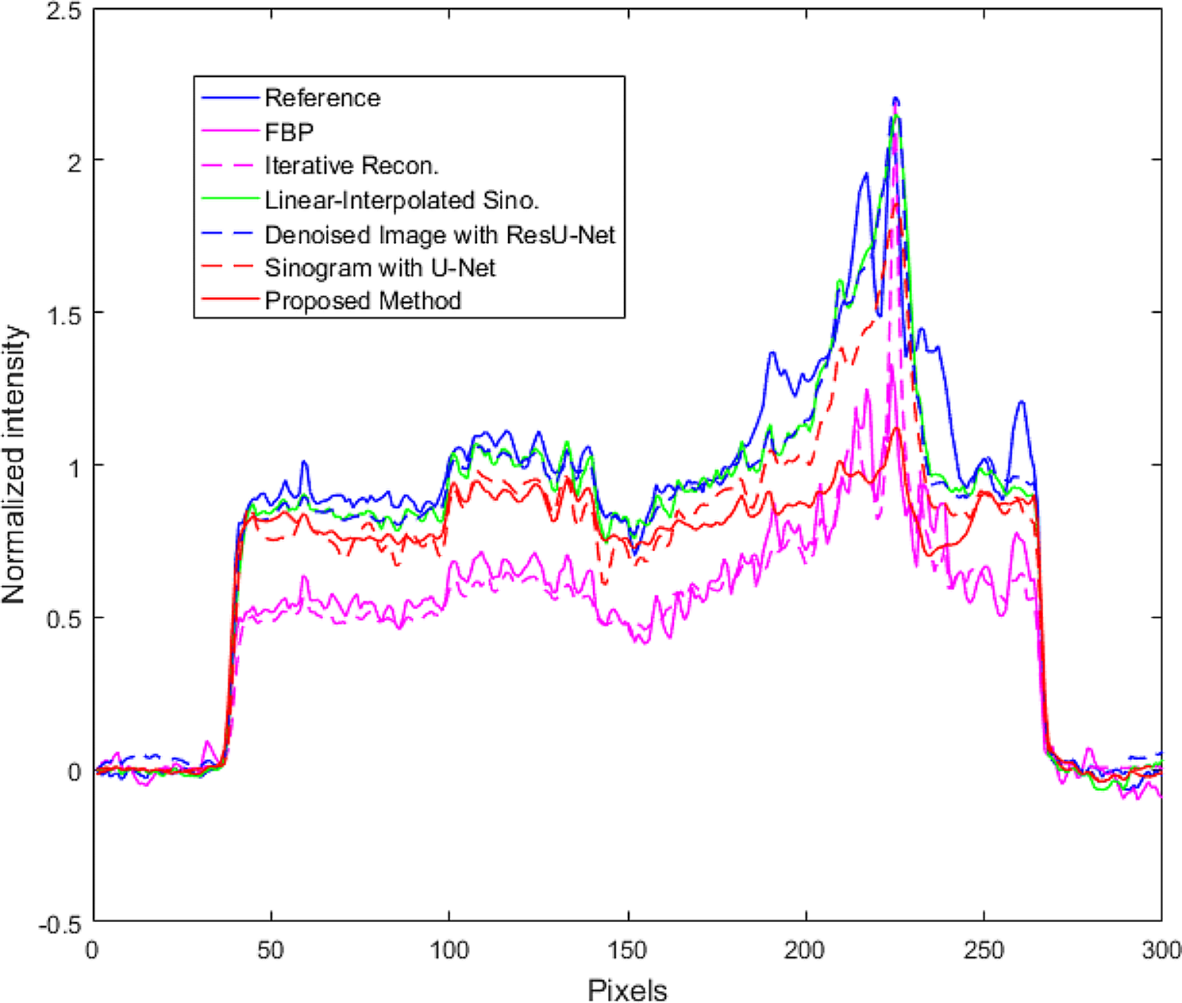
The profiles of the reconstructed images along with the dash-yellow line

The dataset on the second experiment in Group 2 included the breast specimen with a larger marker needle, which generated more metal artifacts and also induced beam hardening [22–23]. Thus, the data near metal objects in the reference image or original images (Fig. 9 (a)) were obliterated by the intensely bright and dark shades. The reconstructed images from FBP and IR are shown in Fig. 9 (b) and (c), respectively. Even though all streaks and metal artifacts were reduced in IR images, they were still easily discernible. Figure 9 (d) depicts the image derived from FBP with linear-interpolated sinograms. Most artifacts on the images using linear-interpolated sinograms were reduced, but some artifacts still appeared in the image (Fig. 9 (d)). For comparison with two deep learning methods, Fig. 9 (e) depicts the denoised FBP image with ResU-Net, and Fig. 9 (f) displays the image from FBP with the sinograms using U-Net. Using the trained U-Net and ResU-Net models, the remaining artifacts on the images (Fig. 9 (f)) can be mitigated; as a result, the improper shades around the marker needle were reduced dramatically. Additionally, some data surrounding metal objects in the images were restored, as indicated by the two red arrows in Fig. 9 (g). Similar to the previous dataset, the quantitative analysis was measured in Table 3. The proposed method using the modified U-Net and ResU-Net models tried to reduce the artifact on the images as provided in the higher SSIM, and the noise from this method was regulated as seen in the increased PSNR. In addition, the CNR values from the proposed method were slightly greater than those derived from other images. Moreover, the profiles were plotted along with a yellow dashed line on the reconstructed image, as shown in Fig. 9 (a). Figure 10 shows that the artifact near the marker needle in the proposed profiles was significantly reduced, and the data away from the needle of the profiles were restored close to those in the reference or original profile.

**Table 3:** Quantitative analysis of image quality and performance of deep-learning models in the reconstructed images from different methods with dataset No. 2 in Group 2

	SSIM	CNR	PSNR
Image from FBP	0.601	2.290	23.654
Image from IR	0.777	5.710	28.208
Image from the sinogram with linear interpolation	0.789	5.210	29.669
Image from denoise with ResU-Net	0.637	5.520	24.640
Image from the sinogram with U-Net	0.856	7.360	29.182
Image from the synthesized sinogram with the modified U-Net and ResU-Net models in the proposed method	0.866	8.330	30.612

## Discussion

During breast conserving surgery, a breast specimen resected from a patient was scanned by micro CBCT to confirm a tumor free margin. To reduce the scan time in micro CBCT, a simple approach is to decrease the number of projections, but this caused degradation in the reconstructed images. Moreover, most breast specimens typically contain a marker metal needle, which causes metal artifacts. This work, therefore, proposed a method for reducing metal and streak artifacts. Here, two stages of neural networks operating on the sinogram and the reconstructed images were added to the traditional technique of reducing metal artifacts (Fig. ;1). The modified U-Net model synthesizes the values in the sinogram, and then modified ResU-Net model deals with the remaining artifacts on the reconstructed images.

The structure of the U-Net model was modified for high performance to synthesize an accurate value in the missing sinogram [7]. While training the U-Net model, the actual details of the sinogram may be lost inside the layers of the model since the model only concentrates on the major features of the input or sinogram. The structure of the U-Net model was forced to undergo the modified down-sampling operation to keep the input details. Here, the convolution technique with the stride of 2 × 2 pixels was used during the down-sampling operation between the kernel and the input to preserve the input details. Additionally, by including the skipping connection from the input layer to the output layer, the convergence of the loss function in the model was accelerated. In Fig. ;4, the validation value was converged and close to the training value. Although the validation value did not additionally approach the training value in the loss function, the trend of convergence and image quality can be acceptable according to their sinogram results.

To evaluate the trained modified U-Net model, Group 2 datasets that were prepared for the reduction of the scan time (down-sampled sinogram) and contained metal data were utilized extensively. The initial metal positions and up-sampled views for the sinograms were determined by linear interpolation, and then the trained model was used to generate the sinogram. As shown in Fig. ;5, the image quality provided by the trained model was superior and comparable to the reference sinogram. As measured by the greater SSIM, the modified U-Net model provided better sinograms than linear interpolation. In terms of error and noise reduction, the MSE and PSNR from the trained model demonstrated superior results, i.e., higher PSNR and lower MSE, compared to those from linear interpolation. Similarly, in Table 2, the Case 2 dataset used in the experiment yielded identical results to Case 1. Nevertheless, the overall experimental results of both cases indicated a high degree of consistency in estimating the correct values when testing the trained model with different datasets. The modified U-net model can train the features in the sinogram domain easier than the image domain due to less complication of the sinogram.

The reconstructed images from the synthesized sinogram and the reference images were used to train the ResU-Net model with the parameters listed in Table 1. The structure of ResU-Net model allowed the input data flowed via connection between stages so that the detailed data in the images were not lost, but a limitation of this model was the slow convergence. However, training the modified ResU-Net model to achieve convergence would take a long time [12] due to many image patches and epochs. Thus, this experiment tries to save training time with an acceptable error of image quality. Having a performance limitation of a personal computer in this study, we compromised the training of the model with available resources and acceptable results.

In the experimental results of the reconstructed images, the proposed method can reduce the main artifacts better than FBP, IR, FBP from the linear-interpolated sinograms, denoised FBP with ResU-Net, and FBP from the sinograms using U-Net. In the case of a small metal needle embedded in the breast specimen, although metal artifacts from the proposed method were reduced as much as those from linear-interpolated sinograms due to a few streaks as shown in Fig. ;7(d) and (g) respectively, the SSIM, CNR, and PSNR appeared higher. In Table 4, the noise on the images of datasets from Group 2 is reduced dramatically as the CNR increased, and the soft tissue detail on the images is significantly restored as the SSIM values increased when compared to other methods. Noise and artifacts such as overshooting (beam hardening effect) in the plotted profiles were significantly reduced by the proposed method, as shown in Fig. 8.

**Table 4:** Quantitative analysis of image quality and performance of deep-learning models in the reconstructed images from different methods with dataset No. 1 in Group 2

	SSIM	CNR	PSNR
Image from FBP	0.025	0.960	6.510
Image from IR	0.382	3.180	15.292
Image from the sinogram with linear interpolation	0.788	7.100	28.055
Image from denoise with ResU-Net	0.816	7.710	28.020
Image from the sinogram with U-Net	0.854	8.010	22.541
Image from the synthesized sinogram with the modified U-Net and ResU-Net models in the proposed method	0.866	8.380	28.726

Another experiment included a breast specimen with a different type of marker needles. The larger the size of the needle, the more beam hardening effect occurs in the reconstructed images and destroys soft tissue data around the marker needle, as shown in Fig. 9 (a). The streak artifacts on the IR images were reduced, but the bright-dark shades around the needle remained dominant (Fig. 9 (c)). However, the reconstructed image from the proposed method performed better than the straightforward FBP, IR and other images using deep learning techniques. Especially, the proposed method not only reduced the bright shade around the marker needle but also significantly restore soft tissue data as two red arrows indicating the improved area (Fig. 9 (g)).

Even though the proposed method tries to address two main artifacts on the reconstructed images, some image details are still not completely recovered because of other causes, such as metal extraction, beam hardening effects. The extraction of metal data from the sinogram based on the segmentation method may be indeed inaccurate; therefore, it is likely that some metal data remain in the sinogram and affect the training efficiency of the model. Another issue is the effect of beam hardening, which is not covered in this work. It may affect the performance of soft tissue restoration since the models of deep learning do not learn the effect of this feature. However, the performance of the proposed method can be further enhanced by retraining in the same ResU-Net model with larger datasets until convergence. Moreover, in this study, we can acquire raw data and all reconstruction parameters from our in-house micro CBCT; however, most commercial CBCT scanners may not permit such access, which may cause some deviated results.

## Conclusions

In this study, we proposed the method for reducing both streak and metal artifacts in breast specimen imaging using deep learning. Four clinical datasets were divided into two groups, and each group contained two breast specimen cases. Group 1 dataset was used to simulate artifacts for training the models, while Group 2 dataset was used for testing. The angular resolution and the metal position of the sparse-view sinograms were improved by deep learning of the modified U-Net model. Here, the synthesized sinograms were reconstructed by the FBP method to obtain the reconstructed images, and the modified ResU-Net model was used to reduce any remaining artifacts on the images. In the experimental results, the overall image quality was more enhanced than the images from the conventional FBP, IR, FBP with the linear-interpolated sinograms, denoised FBP with ResU-Net, and FBP from the sinograms using U-Net. Particularly, both streak and metal artifacts on the images from the proposed method were greatly reduced. Thus, our proposed work to reduce streak and metal artifacts in CBCT reconstructed images and improve breast specimen imaging provided satisfied performance and would be beneficial for clinical use. For future work, improvement of metal extraction and more related artifacts should be covered to further improve image quality.

## Data Availability

The datasets generated and/or analysed during the current study are not publicly available but are available from the corresponding author on reasonable request.

## Abbreviations

CBCT: Cone-beam computed tomography
FBP: Filtered backprojection
IR: Iterative reconstruction
MAR: Metal artifact reduction, ResU-Net:Residual U-Net, RMSE:Root mean error
PSNR: Peak signal-to-noise ratio, ROI:Region of interest
CNR: Contrast-to-noise ratio
SSIM: Structural similarity index

## References

[1] Benjamin W, Maloney DM, McClatchy, Brian W, Pogue KD, Paulsen WA, Wells RJ, Barth (2018). Review of methods for intraoperative margin detection for breast conserving surgery. J Biomed Opt.

[2] Tang R, Buckley JM, Fernandez L (2013). Micro-computed tomography (Micro-CT): a novel approach for intraoperative breast cancer specimen imaging. Breast Cancer Res Treat.

[3] Saowapak S, Thongvigitmanee S, Aootaphao C, Thanasupsombat A, Kiang-ia W, Narkbuakaew K, Wangkaoom P, Junhunee S, Laohawiriyakamol. Puttisak Puttawibul and Pairash Thajchayapong, Cone-Beam CT for Breast Specimens in Surgery: The Phantom Study, 2018 IEEE Nuclear Science Symposium and Medical Imaging Conference Proceedings (NSS/MIC), 2018, pp. 1–3, 10.1109/NSSMIC.2018.8824590.

[4] Brooks RA, Weiss GH, Talbert AJ (1978). A New Approach to Interpolation in Computed Tomography. J Comput Assist Tomogr.

[5] Kostler H, Prummer M, Rude U, Hornegger J. Adaptive variational sinogram interpolation of sparsely sampled CT data. 18th Int Conf Pattern Recognit (ICPR’06). 2006;778–81. 10.1109/ICPR.2006.225.

[6] Li S, Cao Q, Chen Y, Hu Y, Luo L, Toumoulin C. Dictionary learning based sinogram inpainting for CT sparse reconstruction, Optik, volume 125, Issue 12,2014,Pages 2862–2867, ISSN 0030-4026,10.1016/j.ijleo.2014.01.003.

[7] Lee H, Lee J, Kim H, Cho B, Cho S. Deep-neural-network-based Sinogram Synthesis for Sparse-View CT Image Reconstruction. IEEE Trans Radiation Plasma Med Sci. March 2019;3(2):109–19. 10.1109/TRPMS.2018.2867611.

[8] Aootaphao S, Pintavirooj C, Sotthivirat S (2008). Penalized-likelihood reconstruction for metal artifact reduction in cone-beam CT. Annu Int Conf IEEE Eng Med Biol Soc.

[9] Ghani M, Karl WC (2020). Fast enhanced CT metal artifact reduction using data Domain Deep Learning. IEEE Trans Comput Imaging.

[10] Arabi H, Zaidi H (2021). Deep learning-based metal artefact reduction in PET/CT imaging. Eur Radiol.

[11] Ketcha MD, Marrama M, Souza A, Uneri A, Wu P, Zhang X, Helm PA, Siewerdsen JH (2021). Sinogram + image domain neural network approach for metal artifact reduction in low-dose cone-beam computed tomography. J Med Imaging (Bellingham).

[12] Jin Liu Y, Kang J, Qiang Y, Wang D, Chen HY. Low-dose CT imaging via cascaded ResUnet with spectrum loss, methods, Volume 202, 2022, Pages 78–87, ISSN 1046–2023, 10.1016/j.ymeth.2021.05.005. 10.1016/j.ymeth.2021.05.005 33992773

[13] Kyriakou Y, Kalender WA (2007). X-ray scatter data for flat panel detector CT. Phys Med.

[14] Aootaphao S, Thongvigitmanee SS, Rajruangrabin J, Junhunee P, Thajchayapong P (2013). Experiment-based scatter correction for cone-beam computed tomography using the statistical method. Annu Int Conf IEEE Eng Med Biol Soc.

[15] Aootaphao S, Thongvigitmanee SS, Rajruangrabin J, Thanasupsombat C, Srivongsa T, Thajchayapong P (2016). X-ray scatter correction on soft tissue images for portable cone beam CT. Biomed Res Int.

[16] Feldkamp LA, Davis LC, Kress JW (1984). Practical cone-beam algorithm. J Opt Soc Am a.

[17] Kak AC, Slaney M (1988). Principles of Computerized Tomographic Imaging.

[18] Lange K, Carson R (1984). EM reconstruction algorithms for emission and transmission tomography. J Comput Assist Tomogr.

[19] Fessler JA (2000). Statistical image reconstruction methods for transmission tomograph in handbook of medical imaging.

[20] Kim D, Ramani S, Fessler JA (2015). Combining ordered subsets and momentum for accelerated X-ray CT image reconstruction. IEEE Trans Med Imaging.

[21] Aootaphao S, Thongvigitmanee SS, Puttawibul P, Thajchayapong P (2022). Truncation effect reduction for fast iterative reconstruction in cone-beam CT. BMC Med Imaging.

[22] Bayaraa T, Hyun CM, Jang TJ, Lee SM, Seo JK. A Two-Stage Approach for Beam Hardening Artifact Reduction in Low-Dose Dental CBCT, in IEEE Access, vol. 8, pp. 225981–225994, 2020, 10.1109/ACCESS.2020.3044981.

[23] Hsieh J, Molthen RC, Dawson CA, Johnson RH (2000). An iterative approach to the beam hardening correction in cone beam CT. Med Phys.

